# Risk management of hydrogenation station PPP project based on 3D framework—A case study in China

**DOI:** 10.1371/journal.pone.0293348

**Published:** 2023-12-18

**Authors:** Hui Zhao, Guikun Yu, Xian Cheng

**Affiliations:** School of Management Engineering, Qingdao University of Technology, Qingdao, Shandong, China; King Khalid University, SAUDI ARABIA

## Abstract

Renewable hydrogen energy has received growing attention due to the energy shortage and increasing CO_2_ emissions. With these issues in mind, renewable hydrogen has become an important component of future energy systems in many countries, especially in the transportation sector. However, the shortage of hydrogenation station and the risks associated with their construction have become an urgent issue for the development of hydrogen energy transportation. To better implement the hydrogenation station project, a risk management framework is proposed for risk control. First, a comprehensive risk index system is developed, using a weighting method based on the G1 method and the C-OWA operator. Second, a grey fuzzy synthetic assessment method is applied to evaluate the risk based on the 3D risk assessment framework. Finally, risk is assigned to different participants and actionable measures are proposed. This paper summarizes the obstacles to the development of hydrogen energy transportation, highlights the potential of hydrogen energy development, and suggests workable solutions for the use of hydrogen energy in the future transportation industry.

## Introduction

**Fig 1:**
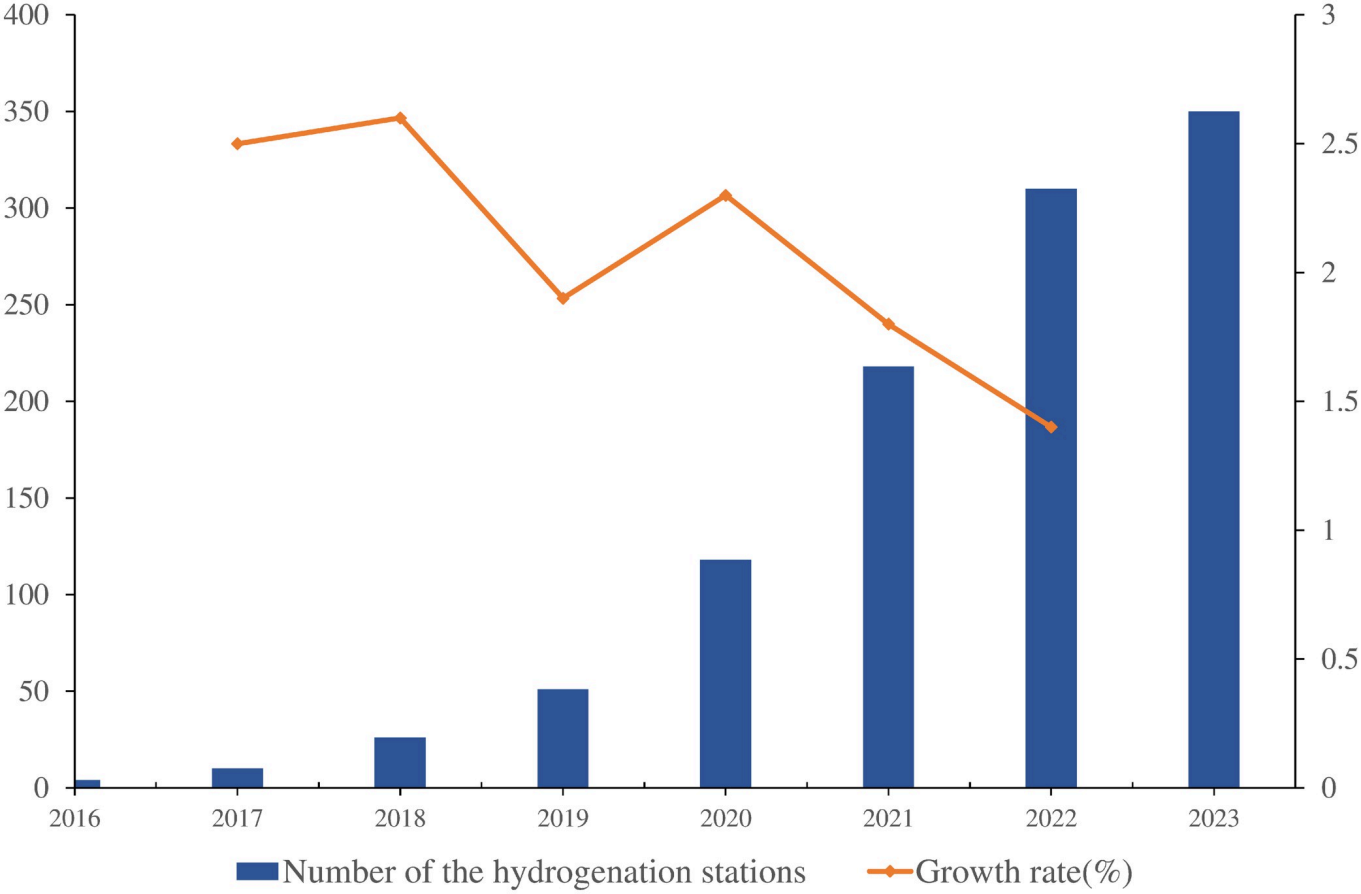
Number of hydrogenation stations built in China over the years.

**Fig 2:**
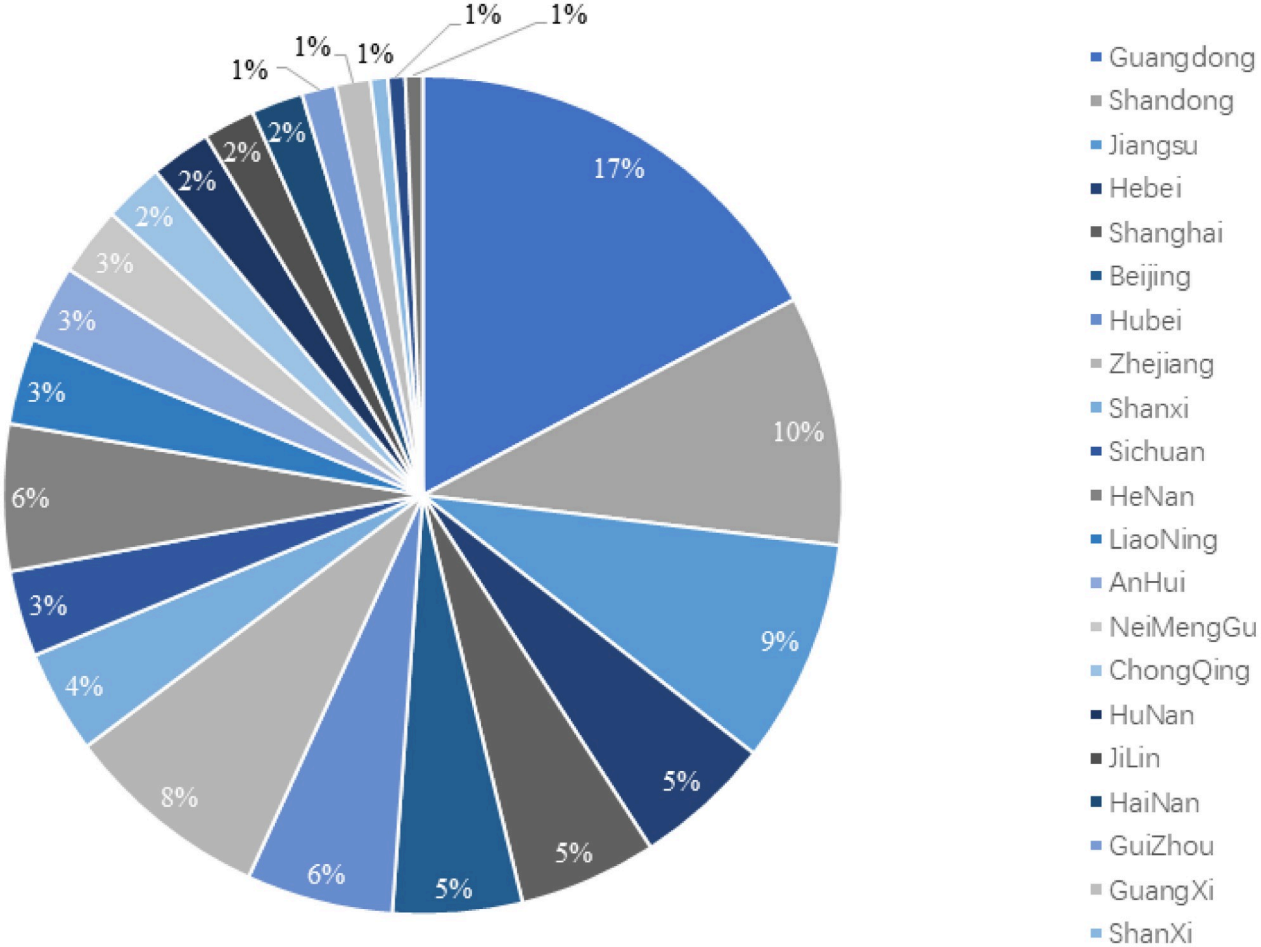
Distribution of hydrogenation stations in China by 2023.

As the drawbacks of fossil fuels in traditional transportation systems are becoming increasingly apparent, hydrogen power has gained broad consensus in China [1]. It aligned with current requirements for the green transformation of China’s transportation industry by promoting reductions in greenhouse gas emissions, increasing energy security, and decreasing local energy consumption [2]. The Chinese government is now considering hydrogen buses as one of the main directions for future urban public transportation and is promoting the use of hydrogen buses in some cities [3, 4]. However, the rollout of hydrogen buses still presents significant challenges in three areas: cost, infrastructure, and technology maturity, with the construction of hydrogenation stations being the most difficult. Although the construction rate of hydrogenation stations in China has increased in recent years (as shown in Fig 1) [5], the overall number is still insufficient, which limits the travel range and usage time of hydrogen buses. In addition, the construction of hydrogenation stations in China also has the problem of uneven distribution. As shown in Fig 2 [6], the current hydrogenation stations are mainly distributed in specific provinces and lack unified planning and layout, which also restricts the promotion and popularity of hydrogen buses.

**Fig 3:**
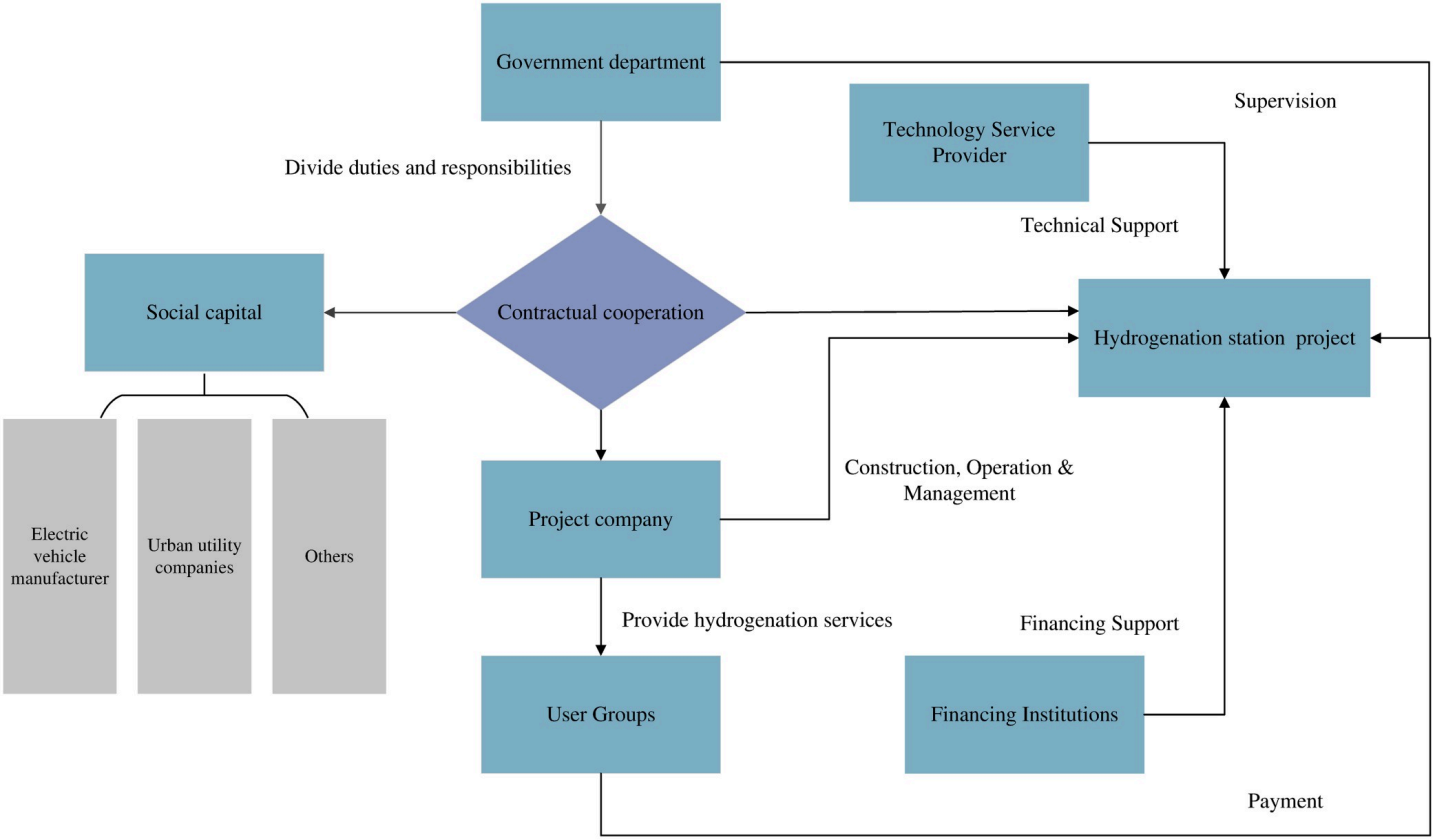
The relationship between the PPP project of the hydrogenation station.

To overcome the above problems and further promote the construction of hydrogen refuelling stations, the Chinese government has adopted a series of Public-Private Partnership(PPP) measures to attract social capital to participate in the construction and operation of hydrogenation stations. The pattern for each stakeholder is shown in Fig 3, which illustrates the collaboration process between the government and private industry to build public infrastructure and deliver public services in a PPP collaboration to construct hydrogen refuelling stations and provide refuelling services. There are four main reasons for applying the PPP mode in the construction of hydrogen stations. Firstly, by adopting the PPP mode, the private sector can participate in the investment and provide financial support, reducing the financial burden on the public sector. Secondly, the private sector typically has extensive operational management experience and expertise to effectively operate and manage hydrogen refuelling station projects. Thirdly, the PPP mode reduces the pressure on the public sector to bear the risk alone. Last but not least, this mode could encourage the private sector to provide more efficient solutions that could help drive the development and adoption of hydrogen refuelling technology.

Although PPP projects have certain advantages over traditional projects, their implementation is often hampered by various risks. At present, many PPP projects have faced such as political instability in regulating water services in Ghanaian [7], delays in road projects in India [8], and financial risks in highway construction projects’ financing [9]. To successfully implement a PPP project for hydrogen energy transportation, a thorough risk management analysis is crucial to identify and mitigate potential risks [10]. In traditional risk assessment, only the uncertainty, loss and uncontrollability of risk are considered, less attention has been paid to the scope of risk impact. And the scope of risk is crucial for PPP projects with many participants. Due to the large number of participants, it is more important to focus on the scope of the risk event, i.e., the range of possible impacts, rather than just on the losses caused by the risk in a hydrogen station construction project based on the PPP model. A framework with the scope of risk as a dimension considers more comprehensively the impacts that a risk event may have on both the government and the private sector in a PPP model. It also helps to identify and assess the multidimensional impacts that may be triggered by a risk event, including government finances, operational management, and corporate reputation.

To address the above problems, this study constructed a hydrogenation station PPP project risk assessment framework from the perspective of three-dimensional based on the probability of occurrence (P), the uncontrollability of the risks (U), and the scope of the risks (S). First, due to a large number of hydrogen refuelling station projects and a large number of experts and scholars conducting relevant research, the Delphi method is adopted to determine the evaluation indicators. After constructing the index system, this study determined the weights of each risk assessment measure using the Lagrangian method combining the subjective G1 method and the objective C-OWA operator method. The G1 method has the advantages of fast calculation, no consistency test, and strong stretching of the number of elements in the same level compared to the AHP. [11–13]. In addition, C-OWA allows us to combine numbers and assign extreme data to low-impact locations and target data to high-impact locations as well as integrate and assign weights to each metric, which greatly reduces the impact of subjective scores and results in more realistic weights that improve the level of application. Further risk assessment based on indicator weights,the use of grey system theory alone cannot completely utilize the fuzziness of assessment criteria, and it has been demonstrated that a single method based on fuzzy mathematics would result in information loss [14]. Consequently,a comprehensive fuzzy gray model is proposed to make full use of these two methods, which provides a feasible method for evaluating the investment risk of hydrogenation complex energy station and makes up for the academic deficiencies in the development analysis of hydrogen energy vehicles. The advantage of the fuzzy gray method is that it not only fully takes into account the fuzziness and grayness of the experts, It also successfully mitigates the impact of subjective elements and prevents the difficulty in assigning weight brought on by too many factors. The research implications of this paper are as follows:

1. Hydrogen refueling station construction project under PPP mode involves multi-party cooperation, and this paper clarifies the risks of each participant, which is very important for the completion of the project.
2. The three-dimensional framework can provide a comprehensive assessment of the risks of hydrogen fuel station projects, and it integrates the principles of sustainable development into the risk management process, which is more applicable to hydrogen refueling station construction projects.
3. This study apply the Lagrangian method to achieve the combination of the advantages of the subjective and objective methods.
4. Proposing solutions that consider the risk distribution and are combined with China’s current situation.

The rest of the paper is organized as follows. Section 2 introduces the current research status of hydrogen energy transportation and the evaluation methods of hydrogen energy transportation. Section 3 describes the risk index system. Section 4 provides a detailed framework for risk management. A case study is presented in section 5. In Section 6, sensitivity analysis is conducted. On account of the results obtained in Section 7, risk mitigation is presented. Finally, conclusions and future research directions are discussed in Section 8.

## Literature review

### Hydrogen energy transportation

Currently, many scholars have studied the current status of hydrogen energy transportation, including its feasibility. Some scholars have also discussed the technical and economic aspects of developing hydrogen energy vehicle and hydrogenation stations. However, there is still a lot of room for researching the risks associated with hydrogen energy vehicles. Many academics are currently investigating the development and viability of hydrogen energy transportation. According to Meng, the hydrogen energy sector in China suffers from high integrated utilization costs,insufficient hydrogen usage norms and regulations, a clear tendency toward blind industrial expansion, and the danger of structural overcapacity. The technical and financial issues of the development of hydrogen vehicle recharging stations have also been covered by several academics. Kovac et al. assert that cost is still a significant element in the advancement of hydrogen energy in China and that novel approaches to hydrogen technology, including its production, storage, delivery, and usage, are infiltrating all industrial sectors [15]. As for the research feasibility and development of hydrogen energy public transport, Thanh Hua et al. discussed some technical indicators of hydrogen fuel cell electric bus (HFCEB), based on the setting and current situation of fuel cell electric bus (FCEB) research and development projects in North America and Europe. It is found that compared with traditional diesel buses, the main obstacle of HFCEB is the lack of refueling infrastructure [16]. O’Garra et al. used a conditional value assessment to compare the public’s capacity to make payments for air and introduced hydrogen buses on a large scale in Berlin, London, Luxembourg to reduce pollution. All cities’ bus riders showed good WTP values for H2 buses, showing support for the buses [17]. Zhu et al. proposed energy stations for large-scale use, and the market is now mostly made up of EV charging energy, but there are some problems [18].

According to Kroniger et al., adding equipment for the production of hydrogen, hydrogen storage, and hydrogenation to charging stations would increase the local consumption of hydrogen energy and increase the effectiveness of the use of renewable energy [19, 20]. In terms of promoting the development of hydrogen energy transportation, Chen et al. proposed that coordination and optimization of operation among subsystems can also reduce operation cost and peak-valley difference of power grid load [21]. El-Taweel et al. proposed a model for optimal scheduling of private hydrogen storage stations that serves both the transportation sector and the electricity market operators, and considered the site selection and operation model of hydrogenation stations based on capacity from the viewpoint of the market [22]. For microgrid operation, Jaramillo et al. indicated a multi-objective mixed integer linear programming model [23]. The car is modeled by Alavi et al. as a community microgrid power plant. Models are used to predict how hydrogen fuel cell vehicles will respond to demand, plan their routes and refill. At the same time, it is proposed that the interaction between the hydrogenation station and the whole system can be further investigated fully [24]. Wu et al. studied the location of clean energy by constructing the best-worst method (BWM) and the multiple attribute boundary approximation region comparison (MABAC) method to study the photovoltaic hydrogen production project, and proposed the location framework [25]. Liao et al. using knowledge measure to obtain weight information, sorted candidate cities of Beijing shared bus stations, and proposed an extended MULTIMOORA method [26]. Rezaei et al. used fuzzy VIKOR to compare wind power stations [27].

### Risk management

In the study of risk management, Wu et al. proposed a risk management framework to control the risk of public-private partnership charging infrastructure for electric vehicles, laying a foundation for the development of electric vehicles [28]. In order to carry out risk identification, Pellegrino et al. believe that public-private joint venture projects need to analyze and allocate a wide range of risks, and divide previous research on Identification of PPP risk into two categories: The risks at the project stage and their nature are the key concerns [29]. Construction and operation risk, microeconomic risk, legal risk, social and political risk, and government risk are the four categories used by Wu et al. to classify hazards.

Risk assessment methods include three well-known methods, namely qualitative assessment, quantitative assessment, and a combination of both [37]. Among the evaluation methods, qualitative evaluation commonly includes expert scoring method [38], quantitative evaluation mainly includes decision tree analysis and the Monte Carlo simulation approach [39]. Han et al. take qualitative and quantitative approaches to study the risk of urban natural gas pipeline networks. The quantitative method consists of a probability assessment, a consequences analysis and arisk evaluation. The outcome of the qualitative method is a qualitative risk value, and for quantitative method the outcomes are individual risk and social risk. [40] Qualitative and quantitative evaluation methods include gray evaluation approach, fuzzy comprehensive evaluation method, and analytical hierarchy process [41–43]. The advantages and disadvantages of these methods and their characteristics are summarized in Table 1.

**Table 1:** Comparison of different types of risk assessment methods.

Method	Advantages	Disadvantages
Qualitative method [30–33]	Easy to use, comprehend and accomplish	Subjective biases in processes and measurements
	Save time and cost	Lack of distinction in the key dangers of effect analysis
Quantitative method [33–35]	Widely applicable	Complex and lengthy procedure
	Based on an objective procedure	Sporadically obtainable data
	With a reasonably accurate outcome	Reliance on mathematical models
Comprehensive method [33, 36]	Combination of subjective and objective methods	More difficult to deploy
	High accuracy	More sophisticated and requiring more readily available data

The changing dynamics of risk management in PPP projects reflects how risk changes over time. Most studies are based on two dimensions of risk level acquisition. Xu and Wu et al. took capacity and severity respectively into consideration in the study of risk ranking, as well as the possibility of occurrence and the magnitude of impact [10, 44]. Participants were assigned the risks of Colombia’s social infrastructure based on several risk categories by Sastoque et al. [45]. Artificial neural networks were used by Jin and Zhang [46] to optimize risk allocation. Abednego and Ogulana [47] provide a case study of a turnpike project. In this case, the risk allocation is mainly achieved for a PPP toll road project to achieve project governance. Through a case study of the Sydney rail PPP project, Ng and Loosemore [48] revealed the rationale for risk distribution choices between the public and private sectors.

## Establishment of risk index system

**Fig 4:**
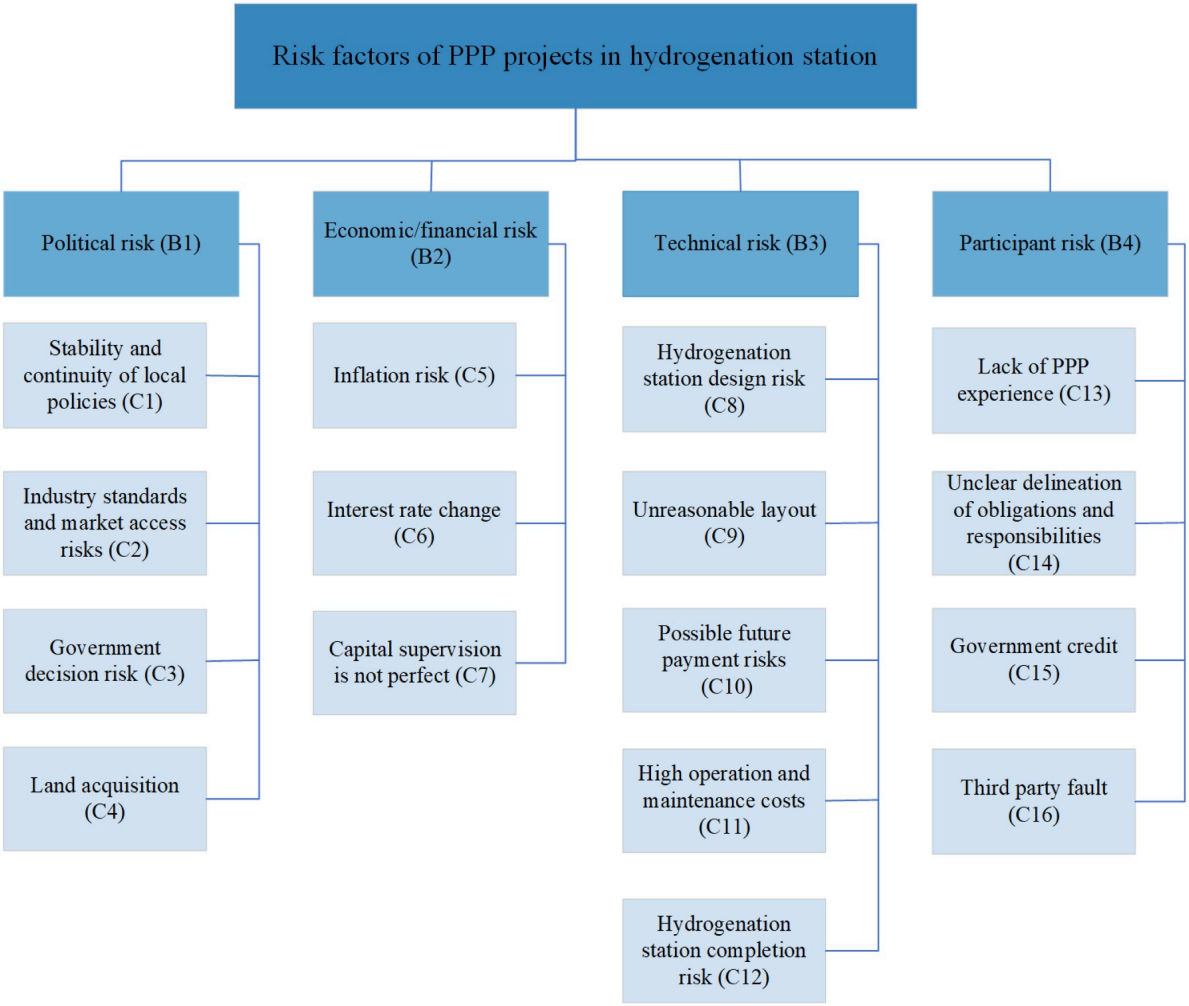
Risk factors of hydrogenation station construction.

Risk indexing systems play a crucial role in risk management and have a direct impact on the accuracy and effectiveness of the findings. During the establishment process, it is important to consider risks from various perspectives. In addition to the potential losses caused by the uncertainty of the investment process, we should comprehensively consider the investment risks of relevant overseas projects, the market development situation, and the significant differences in laws and regulations. To accomplish this, we enlisted the help of key specialists in the fields of risk management, PPP models, and new energy vehicles to identify the risk variables through the Delphi approach. Based on the particularity of hydrogenation station technology and the characteristics of PPP projects, this paper selects experts from universities, new energy enterprises and relevant government departments who have studied PPP projects for the purpose of low-carbon research. The selected experts have the following requirements: Professors from well-known universities with more than 3 years of research experience in PPP project or new energy project construction; Experts actively involved in government agencies and private capital projects; Professionals who have worked in large new energy project construction companies and participated in several new energy projects; Lawyers from well-known law firms to provide legal advice to hydrogenation energy station PPP project; And government officials responsible for promoting new energy projects. Details of the selection of experts are shown in Table 2. Objectives were then identified and a detailed outline of the questions to be answered by the experts was developed, along with relevant background material to be provided to the experts. 16 risk factors—divided into four categories by political risk, financial risk, technical risk of building the hydrogenation station, and participant acquisition risk—are ultimately identified through analysis and discussion of the risks that could cause the PPP hydrogenation station project to fail. Detailed risk factors are shown in Fig 4.

**Table 2:** Information of the experts.

No	Work unit	Position
Expert#1	Tsinghua University	Professor
Expert#2	Chongqing University	Professor
Expert#3	Qingdao City Financing Department	Official
Expert#4	Qingdao Energy Co. LTD	Chief engineer
Expert#5	China International Engineering Consulting Corporation	Professional advisor
Expert#6	Ning De Times New Energy Technology Co. LTD	Professional advisor
Expert#7	Zhong Lun Law Firm	Lawyer
Expert#8	Beijing environmental protection Department	Official
Expert#9	Qingdao Public Transport Group Co. LTD	General manager
Expert#10	Qingdao Port International Co. LTD	Technical manager

### Political risk(B1)

The political risk includes that those posed by governmental regulations and the hydrogen-powered automobile sector itself, are concentrated in the political and industrial spheres. These risks directly affect the construction conditions and direction of hydrogenation facilities on a macro level.

#### Stability and continuity of local policies(C1)

It refers to the potential impact that changes in local policies, regulations, or laws can have on the operation, profitability, and viability of a hydrogenation station. This risk can arise from a range of factors, such as changes in zoning regulations, land-use policies, or permit requirements related to the construction or operation of hydrogenation stations [49, 50].

#### Industry standards and market access risks(C2)

By referring to foreign experience and combining with the existing related standards and specifications in our country, such as “Technical specification for Gas filling Station for automobile”, “Code Design for Hydrogen and Oxygen Station”, etc., to ensure the inheritance and compatibility of the standards and specifications established to the maximum extent, also inevitable existence [51–53].

#### Government decision risk(C3)

Government departments at various levels need to make decisions for the infrastructure project of hydrogenation station. Complicated implementation procedures could have an adverse effect on the project. Meanwhile, a decision process that lacks standards and is poorly prepared can lead to wrong decisions [54].

#### Land acquisition(C4)

The infrastructure for hydrogenation needs more planning, which puts this vulnerability at risk [48, 49, 51, 53].

### Economic/Financial risks(B2)

For such indexes, changes in the micro financial environment and the ongoing availability of project funding are the two key sources of risk.

#### Inflation risk(C5)

This might negatively impact how the economy typically operates, usually accompanied by price increases, such as basic hydrogen storage and transportation equipment, it could have an impact on the profitability and cost of building hydrogen filling stations [45, 55].

#### Interest rate change(C6)

The PPP hydrogenation station facility project’s finance costs and project value will be impacted by changes in interest rates during development and operation [53].

#### Capital supervision is not perfect(C7)

High investment PPP projects involve many participants, which leads to regulatory difficulties in the system of risk indicators for the hydrogenation station infrastructure. This risk may lead to the interruption of funding, which is likely to lead to the termination of the project [51, 56].

### Technical risks(B3)

#### Hydrogenation stations design risks(C8)

It refers to the potential impact that design choices can have on the safety, efficiency, and reliability of a hydrogenation station. These risks can arise from a variety of factors, such as the selection of equipment, the layout of the station, the choice of materials, and the implementation of safety measures [57].

#### Unreasonable layout(C9)

There is a risk that hydrogenation station facilities will be left idle and underutilized to their full potential due to poor layout. This risk is directly related to revenue, as tariffs are one of the main sources of revenue [2, 46].

#### Possible future payment risks(C10)

For payment processing, third party payments are not yet effectively used in the area of toll infrastructure. And the possible presence of information security can be a barrier to the use of hydrogenation infrastructure [58].

#### High operation and maintenance costs(C11)

This risk is caused by the still labour-intensive mode of operation and maintenance of hydrogen refuelling infrastructure and the current incomplete technology for the construction of hydrogen refuelling facilities [46, 48, 51, 59].

#### Risk of hydrogenation station completion(C12)

This is primarily due to the fact that the current hydrogen supply chain system is ineffective and that hydrogen production, storage, and transportation do not make up an entire and effective supply chain system. There are a number of uncertainties facing the completion of the hydrogenation stations [18].

### Participant risk(B4)

PPP models frequently incorporate more than two participants, including the public and private sectors as well as contractors. Therefore, the risks posed by these participants themselves are inevitable.

#### Lack of PPP experience(C13)

As a new model in PPP hydrogenation infrastructure projects, risks may arise due to the lack of overall planning experience of the participants. For example, Difficulties in framing the terms of the contract [28, 44, 55].

#### Unclear delineation of obligations and responsibilities(C14)

This risk means that as PPP projects involve multiple players, so the boundaries of duty and obligation can be hard to delineate [51, 53, 55].

#### Government Credit(C15)

In PPP to infrastructure projects, being completely fair in both the public and private spheres is challenging. The government sector can use its own public rights resources to counteract the execution of project contracts that lead to project failure [46, 55].

#### Third party default(C16)

Multiple participants in a PPP project cause the project to be delayed or to fail without any of the participants’ fault [46, 51].

## Methdology

**Fig 5:**
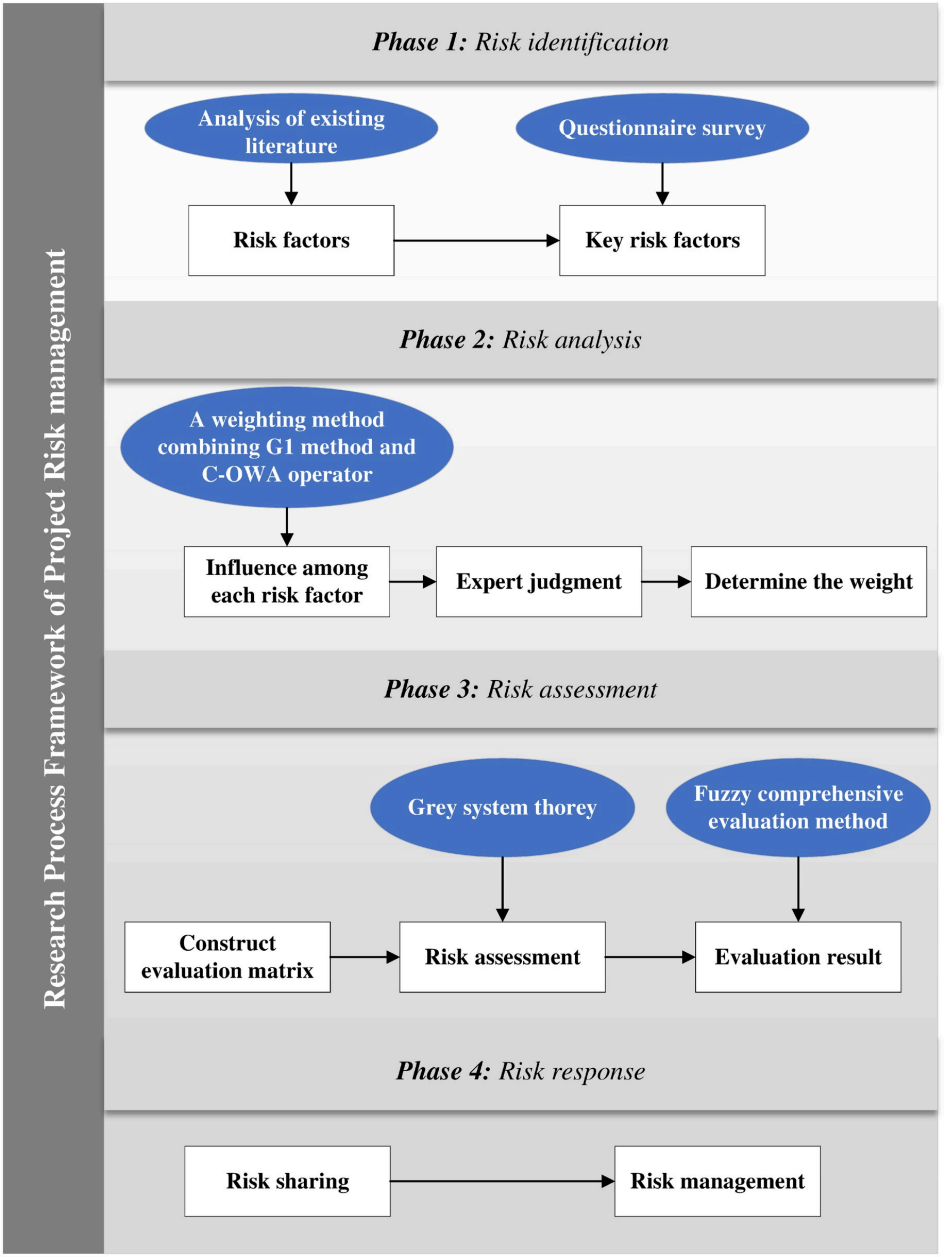
Risk management process.

Effective risk management is crucial to achieving win-win situations. Firstly, the comprehensive risk indicator system is established by using the Delphi method. Secondly, the 3D model including probabilities, scopes, and uncontrollability is applied for risk assessment, where the weight is determined by using the G1 method and an assignment method that combines C-OWA operators and a gray blur method for evaluation. Finally, each participant is assigned different risks. The detailed process is shown in Fig 5.

### Empowerment method based on a combination of the G1 method and C-OWA

Based on the requirements of comprehensiveness, comparability, systematics and science, the Delphi method was applied to classify the risks of hydrogenation station PPP projects into four primary factors and 16 secondary factors. The combination weighting method was used to determine the weight of each key risk factor and the different levels of influence of each risk factor in the hydrogenation station PPP project. Prior to risk management, after the risk assessment indicator system for a hydrogenation station PPP project has been identified, the precise weighting of each indication must be determined using a suitable approach.

Currently, the combined weighting method is becoming a trend [60]. In this paper, the weights of each risk evaluation indicator are determined using the combined weighting approach. The G1 approach, which relies on subjective expert ratings, produces extreme values that do not reflect changes in objective conditions. However, decision data may be combined with C-OWA. By combining numbers, assigning extremes to locations with low influence and target data to locations with high influence. It mitigates the influence of subjective scoring that can produce extreme values and takes the weights closer to the true nature of reality. As a result, the two methods together prevent the subjective and objective bias that one strategy may bring about.

#### Subjective weighting based on the G1 method

The AHP approach separates the problem into multiple levels and components, assesses the level of importance between the two indicators, creates a judgment matrix, computes the eigenvalue and associated eigenvector of the judgement matrix, and then determines the weight that should be applied. Based on AHP, Guo introduced the G1 method, which is an enhanced subjective weighting approach based on expert judgement. It is worth noting that the order of importance of a variable does not affect its ranking.

Step 1: Rank the risks according to their importance. Assume that the set of risk factors is *C* = (*c*_1_, *c*_2_, …, *c*_*n*_), if c_*l*_ (*lϵ*1, 2, ⋯, *n*) is more important than c_*k*_(*kϵ*1, 2, ⋯, *n*),then it is *c*_*l*_>*c*_*k*_. The comparison between the other indicators is similar, and thus the importance ranking of each indicator is obtained.

Step 2: Determine relative importance. According to the experts in Table 2 determine the adjacent risk factors *c*_*i*_ and *c*_*i*−1_ of *r*_*i*_ = *c*_*i*−1_/*c*_*i*_. The *r*_*i*_ assignments are shown in Table 3.

**Table 3:** The value of relative importance.

Value	Description
1.0	*C*_*i*−1_ has the same significant as *C*_*i*_
1.2	*C*_*i*−1_ is somewhat more significant than *C*_*i*_
1.4	*C*_*i*−1_ is more significant than *C*_*i*_
1.6	*C*_*i*−1_ is far more significant than *C*_*i*_
1.8	*C*_*i*−1_ is extremely more significant than *C*_*i*_

Step 3: By utilizing Eqs 1 and 2, we can determine the subjective importance of each risk factor:

$$\begin{array}{c} \omega_{i}=\left(1+\sum_{i=2}^{n}\prod_{i}^{n}r_{i}\right) \end{array}$$ (1)

$$\begin{array}{c} \omega_{i-1}=\omega_{i}*r_{i} \end{array}$$ (2)

Given that the experts listed in Table 2 have voting rights, the final weights are determined through a calculation process that considers the decision makers’ weighting according to the following equation:

$$\begin{array}{c} \omega_{i}^{\prime }=\frac{\omega_{1i}+\omega_{2i}+\cdots +\omega_{pi}}{p} \end{array}$$ (3)

where *ω*_*pi*_ is calculated as the product of the weight of the indicator relative to the criterion layer and the weight of the criterion layer.

#### Objective weights using the C-OWA operator

The theory of the OWA operator was first proposed by Professor Yager [61]. Academics have proposed different types of OWA operators. The C-OWA operator has been extensively utilized to compute indicator weights since it is an ordered weighted average operator depending on the number of combinations [62]. The steps are as follows:

Step 1: The initial scoring dataset for each expert *B* = {*b*_1_, *b*_2_, ⋯, *b*_*n*_}, the scoring data were sorted from largest to smallest and numbered starting from 0. The results were obtained *b*_0_ ≥ *b*_1_ ≥, ⋯, ≥ *b*_*j*_ ≥, ⋯, *b*_*m*_.

Step 2: By combining numbers $C_{n-1}^{j}$, directly determine the weight *ω*_*i*+1_ of the data *θ*_*j*+1_,

$$\begin{array}{c} \omega_{i+1}=\frac{C_{n-1}^{j}}{\sum_{k=0}^{n-1}C_{n-1}^{k}}=\frac{C_{m}^{j}}{2^{n-1}},\;j=0,1,\ldots ,n-1 \end{array}$$ (4)

Step 3: Passing the weights *θ*_*j*+1_, the initial decision dataset is success fully *Z* is weighted and the absolute weight ${\bar{\omega }}_{i}$ of the index is calculated by the following equation:

$$\begin{array}{c} {\bar{\omega }}_{i}=\sum_{j=0}^{n-1}\theta_{j+1}b_{j},\; \end{array}$$ (5)

where n indicates the number of indicators.

Step 4: By using the following Equation, based on the index *C*_*i*_, calculate the relative weight value $\omega_{i}^{\prime \prime }$:

$$\begin{array}{c} \omega_{i}^{\prime \prime }=\frac{{\bar{\omega }}_{i}}{\sum_{j=0}^{n-1}\omega_{i}},\; \end{array}$$ (6)

#### Combination weight based on the Lagrangian

The final risk factor weights are more scientifically justified thanks to the combined weighing approach, which combines the G1 method with the C-OWA operator, incorporates the expert’s professional judgement into the G1 method, and employs the entropic objective weighting method to prevent subjectivity. Given that subjective weight $\omega_{i}^{\prime }$ and objective weight $\omega_{i}^{\prime \prime }$(*i* = 1, 2, 3, ⋯, *m*), a Lagrangian theory-based risk factor combination weight optimisation model is established, the combination weight model is shown as follows:

$$\begin{array}{c} \omega_{i}=v_{1}\omega_{i}^{\prime }+v_{2}\omega_{i}^{\prime \prime }, \end{array}$$ (7)

where *v*_1_ and *v*_2_ are the coefficients to be determined, and the undetermined coefficients *v*_1_ and *v*_2_ can be transformed into the following optimization problem:

$$\begin{array}{c} \left\{ \begin{array}{c} \text{max}\;F(v_{1},v_{2})=\sum_{i=1}^{m}(\sum_{j=1}^{n}(v_{1}\omega_{i}^{\prime }+v_{2}\omega_{i}^{\prime \prime })),\\ v_{1},v_{2}\geq 0,v_{1}+v_{2}=1 \end{array}\right. \end{array}$$ (8)

In addition, depending on the Lagrangian extremum condition, the Lagrangian function can be used to *v*_1_ and *v*_2_,as shown in Eqs 9 and 10:

$$\begin{array}{c} \left\{ \begin{array}{c} v_{1}^{\prime }=\frac{\sum_{i=1}^{m}\sum_{j=1}^{O}\omega_{i}^{\prime }x_{ij}}{\sqrt{{\left(\sum_{i=1}^{m}\sum_{j=1}^{n}\omega_{i}^{\prime }x_{ij}\right)}^{2}+{\left(\sum_{i=1}^{m}\sum_{j=1}^{n}\omega_{i}^{\prime \prime }x_{ij}\right)}^{2}}}\\ v_{2}^{\prime }=\frac{\sum_{i=1}^{m}\sum_{j=1}^{n}\omega_{i}^{\prime \prime }x_{ij}}{\sqrt{{\left(\sum_{i=1}^{m}\sum_{j=1}^{n}\omega_{i}^{\prime }x_{ij}\right)}^{2}+{\left(\sum_{i=1}^{m}\sum_{j=1}^{n}\omega_{i}^{\prime \prime }x_{ij}\right)}^{2}}} \end{array}\right. \end{array}$$ (9)

$$\begin{array}{c} \left\{ \begin{array}{c} v_{1}=\frac{v_{1}^{\prime }}{\left(v_{1}^{\prime }+v_{2}^{\prime }\right)}\\ v_{2}=\frac{v_{2}^{\prime }}{\left(v_{1}^{\prime }+v_{2}^{\prime }\right)} \end{array}\right. \end{array}$$ (10)

### Grey fuzzy comprehensive evaluation

**Fig 6:**
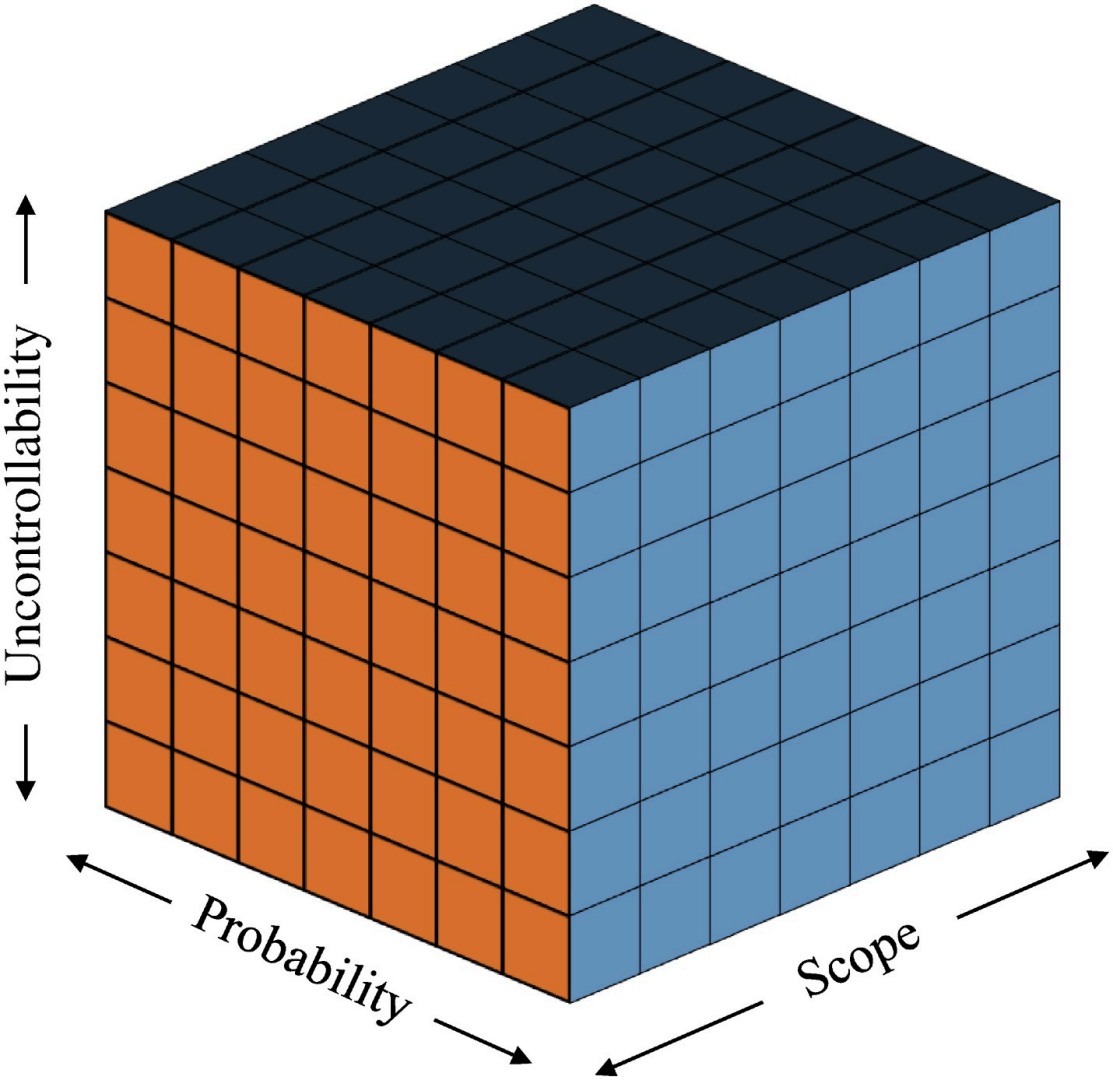
3D risk assessment framework.

A paradigm for three-dimensional risk assessment is suggested in this research (as shown in Fig 6). The experts scored the risks in 3 dimensions and the final results were obtained from $R=\sqrt[{3}]{P*S*U}$. Given that the PPP hydrogenation station infrastructure project’s risk index system exhibits fuzzyness and ambiguity, which is consistent with its gray features, the grey fuzzy comprehensive evaluation method was used to assess the project.

Step1. Create a sample matrix.

Assume that three perspectives P, S and U are used to evaluate risk by five experts. The assessment of risk made by the k^*th*^ expert is stated as *d*_*ki*_. Following that, a sample matrix D is produced using the information supplied by the experts for all risk indicators.

$$\begin{array}{c} D=[\begin{array}{cccc} d_{11} & d_{12} & \cdots & d_{1n}\\ d_{21} & d_{22} & \cdots & d_{2n}\\ \vdots & \vdots & \vdots & \vdots \\ d_{p1} & d_{p2} & \cdots & d_{pn} \end{array}] \end{array}$$ (11)

Step2. Determine the grey level of the assessment.

The risk assessment of the PPP hydrogenation infrastructure is divided into 5 levels, Consistent values are 9, 7, 5, 3 and 1, with values of 8, 6, 4 and 2 between the two levels. The whitening weighting function is shown below.

$$\begin{array}{c} f_{1}(d_{ki})=\{\begin{array}{cc} 0, & d_{ki}\notin \left[0,10\right]\\ \frac{1}{9}d_{ki}, & d_{ki}\in \left[0,9\right)\\ 1, & d_{ki}\in \left[9,10\right] \end{array}, \end{array}$$ (12)

$$\begin{array}{c} f_{2}(d_{ki})=\{\begin{array}{cc} 0, & d_{ki}\notin \left[0,10\right]\\ \frac{1}{7}d_{ki}, & d_{ki}\in \left[0,7\right)\\ \frac{10-d_{ki}}{3}, & d_{ki}\in \left[7,10\right] \end{array}, \end{array}$$ (13)

$$\begin{array}{c} f_{3}(d_{ki})=\{\begin{array}{cc} 0, & d_{ki}\notin \left[0,10\right]\\ \frac{1}{5}d_{ki}, & d_{ki}\in \left[0,5\right)\\ \frac{10-d_{ki}}{5}, & d_{ki}\in \left[5,10\right] \end{array}, \end{array}$$ (14)

$$\begin{array}{c} f_{4}(d_{ki})=\{\begin{array}{cc} 0, & d_{ki}\notin \left[0,10\right]\\ \frac{1}{3}d_{ki}, & d_{ki}\in \left[0,3\right)\\ \frac{10-d_{ki}}{7}, & d_{ki}\in \left[3,10\right] \end{array}, \end{array}$$ (15)

$$\begin{array}{c} f_{5}(d_{ki})=\{\begin{array}{c} 0,\;d_{ki}\notin \left[0,10\right]\\ 1,\;d_{ki}\in \left[0,1\right)\\ \frac{10-d_{ki}}{9},\;d_{ki}\in \left[1,10\right] \end{array} \end{array}$$ (16)

Step3. Build the grey evaluation matrix.

According to the above formulas, we can get the grey statistics *g*_*ij*_ and the total grey statistic denoted as *G*_*i*_ of the total grey statistics, as follows:

$$\begin{array}{c} g_{ij}=\sum_{k=1}^{n}f_{e}(d_{ki}) \end{array}$$ (17)

$$\begin{array}{c} G_{i}=\sum_{j=1}^{M}g_{ij} \end{array}$$ (18)

The gray level can be calculated using the above two formulas, which are represented as follows:

$$\begin{array}{c} r_{ij}=\frac{g_{ij}}{G_{i}} \end{array}$$ (19)

Therefore, the calculation of the grey fuzzy evaluation matrix can be performed as follows:

$$\begin{array}{c} R=[\begin{array}{cccc} r_{11} & r_{12} & ... & r_{1s}\\ r_{21} & r_{22} & ... & r_{2s}\\ \vdots & \vdots & \vdots & \vdots \\ r_{M1} & r_{M2} & .. & r_{Ms} \end{array}] \end{array}$$ (20)

Step4. Fuzzy overall assessment.

We can obtain the final evaluation results after creating the matrix. There are two extensive evaluation levels in this area. First, the following Equation may be used to compute the first level comprehensive assessment matrix, designated as *B*.

$$\begin{array}{c} B_{i}=W_{i}{}^{\circ }R_{i}=(b_{i1},b_{i2},\cdots ,b_{in}),i=1,2,\cdots ,N \end{array}$$ (21)

Consider *C*_*i*_ as a single factor, then use the Level 1 composite assessment’s findings as a single factor evaluation. Based on this, it may contain a total evaluation matrix, denoted by the letter *M*:

$$\begin{array}{c} M=[\begin{array}{c} B_{1}\\ B_{2}\\ \vdots \\ B_{N} \end{array}]=[\begin{array}{cccc} b_{11} & b_{12} & \cdots & b_{1n}\\ b_{21} & b_{22} & \cdots & b_{2n}\\ \vdots & \vdots & \vdots & \vdots \\ b_{N1} & b_{N1} & \cdots & b_{Nn} \end{array}] \end{array}$$ (22)

Finally, we obtained the second level *B* = *W* ∘ *R* = (*b*_1_, *b*_2_, ⋯, *b*_*N*_) of the integrated assessment matrix. Finally, using the Eq 23 for a five-point system (1 = extremely low, 3 = low, 5 = medium, 7 = high, and 9 = very high), the composite risk value *Z* can be calculated.

$$\begin{array}{c} Z=B•V^{T}=B•{\left(9,7,5,3,1\right)}^{T} \end{array}$$ (23)

## Case study

Before conducting risk management, this study employs a combined G1 method and C-OWA method to calculate the precise weights of each index after identifying the risk assessment index system of the hydrogenation station PPP project. The subjective weighting method, although capable of reflecting experts’ attention to various factors, possesses limitations due to its heavy reliance on expert opinions. On the other hand, while objective weighting can be effective in exploiting the quality of the data itself, it neglects the human side of decision-making, making it less practical. Therefore, a joint weighting approach can enhance constraints. First, the raw risk data is undimensional. Based on the experience and knowledge of the relevant experts, the 10 experts, the original risk data (listed in S1 File) is obtained by assigning values to each risk factor (as shown in Table 4) according to their sensitivities. To achieve uniformity, a range analysis dimensionless matrix,denoted as F,was generated as shown in S2 File.

**Table 4:** Risk sensitivity.

Risk sensitivity	Corresponding value range
Very high (x1)	[0.8,1.0)
High (x2)	[0.6,0.8)
Moderate(x3)	[0.4,0.6)
Low(x4)	[0.2,0.4)
Very Low(x5)	[0,0.2)

Next, the weights for C1 to C16 were determined using the combined weights method. Sort the data according to the original expert scoring data, taking indicator C1 as an example, the number of experts and n are 10. Bringing the data into the MATLAB code of Eqs 4 and 5, the absolute weight of the calculated indicator can be obtained. Finally, according to the MATLAB calculation of Eq 6 can be obtained to calculate the objective weight of the indicator. while the subjective weights are calculated using the G1 approach described in the Equation. Finally, according to Eqs 8–10, the coefficients V1 and v2 can be optimized by the Lagrangian extremum condition, which are 0.5 and 0.5, respectively. After the value of v1,v2 is obtained, the final combined weight can be calculated according to Eq 7. The relevant results are shown in Table 5.

**Table 5:** Weight result.

First-level risk factors	Weight	Second-level risk factors	Subjective weight	Objective weight	Combination weight
B1	0.218	C1	0.061	0.066	0.064
		C2	0.061	0.065	0.063
		C3	0.056	0.053	0.054
		C4	0.039	0.038	0.038
B2	0.183	C5	0.019	0.021	0.020
		C6	0.116	0.119	0.118
		C7	0.046	0.042	0.044
B3	0.342	C8	0.122	0.126	0.124
		C9	0.107	0.104	0.105
		C10	0.039	0.038	0.039
		C11	0.043	0.040	0.041
		C12	0.031	0.036	0.034
B4	0.257	C13	0.027	0.031	0.029
		C14	0.157	0.153	0.155
		C15	0.033	0.042	0.038
		C16	0.038	0.043	0.041

Based on the results of the combination weights, we can obtain the weighting of the risk factors for the B-level and C-level indicators. Each risk factor was scored by five experts from the risk domain and the clean energy domain based on three risk assessment dimensions, as shown in Table 6. The five experts from Table 1 are Expert 1, Expert 2, Expert 6 for the risk domain and Expert 4 and Expert 7 for the clean energy domain. The combined risk values were then normalized and the results are shown in Table 7, where $A=\sqrt[{3}]{P*S*U}$. C2 (industrial standards and market access risk) ranked first for all risk factors, and it is worth noting that C1 (stability and continuity of local policies), which scored the highest, should have measures taken in advance to minimise losses. For participants, the focus should be on key risk factors with standardised values equal to or greater than 0.50. These risk factors include C2 (industry standards and market access risk), C1 (stability and continuity of local policies), C13 (lack of PPP experience), C8 (hydrogenation station design risk), C14 (unclear delineation of responsibilities and obligations), C13 (lack of PPP experience) and C11 (high operation and maintenance costs).

**Table 6:** Initial scores.

Risk factors	P					S					U				
C1	8	6	9	6	6	5	6	7	7	6	8	9	8	8	8
C2	8	7	9	8	6	8	8	6	8	7	7	8	6	6	6
C3	5	2	4	3	3	4	6	5	5	5	5	4	5	5	4
C4	4	5	4	4	5	3	2	2	4	5	5	3	5	4	3
C5	6	6	5	6	5	3	4	4	4	5	3	4	5	3	3
C6	7	6	5	5	4	4	5	4	4	3	3	4	4	2	4
C7	3	3	4	4	3	4	3	3	4	4	2	3	4	3	3
C8	8	6	8	7	5	7	6	5	5	5	6	3	4	6	6
C9	7	6	6	5	5	6	6	5	6	5	4	4	4	5	6
C10	5	4	5	6	5	5	6	5	6	5	4	4	3	3	4
C11	7	6	6	5	6	5	6	4	5	5	5	4	3	4	6
C12	6	4	5	5	4	4	4	4	5	4	4	4	4	5	6
C13	6	6	8	6	5	7	8	5	5	5	6	6	6	6	7
C14	6	5	8	5	6	8	8	6	3	5	5	5	6	5	5
C15	3	3	5	2	4	2	3	4	3	2	2	2	3	2	3
C16	4	4	6	5	4	4	5	4	3	3	5	5	4	3	5

**Table 7:** Ranking of the risk factors.

	P	S	U	A	Normalization	Ranking
C2	7.6	7.4	6.6	7.19	1.00	1
C1	7.0	6.2	8.2	7.09	0.97	2
C13	6.2	6.0	6.4	6.21	0.77	3
C8	6.8	5.6	5	5.75	0.67	4
C14	6.0	6	5.2	5.72	0.66	5
C9	5.8	5.6	4.6	5.31	0.56	6
C11	6.0	5	4.4	5.09	0.51	7
C10	5.0	5.4	3.6	4.60	0.4	8
C12	4.8	4.2	4.6	4.53	0.38	9
C5	5.6	4.0	3.6	4.32	0.33	10
C3	3.4	5.0	4.6	4.28	0.32	11
C6	5.4	4.0	3.4	4.19	0.3	12
C16	4.6	3.8	4.4	4.25	0.32	13
C4	4.4	3.2	4.0	3.83	0.22	14
C7	3.4	3.6	3.0	3.32	0.10	15
C15	3.4	2.8	2.4	2.84	0.00	16

According to Eqs 11–21, a grey fuzzy evaluation matrix can be obtained.

Then the evaluation matrix M by Eq 22 can be obtained as follows:

$$\begin{array}{c} M_{P}=[\begin{array}{ccccc} 0.2144 & 0.2145 & 0.1996 & 0.1967 & 0.1748\\ 0.1593 & 0.1853 & 0.2276 & 0.2278 & 0.2000\\ 0.2038 & 0.2340 & 0.2412 & 0.1806 & 0.1404\\ 0.1873 & 0.2287 & 0.2166 & 0.1964 & 0.1711 \end{array}] \end{array}$$ (24)

$$\begin{array}{c} M_{S}=[\begin{array}{ccccc} 0.1958 & 0.2335 & 0.2332 & 0.1861 & 0.1515\\ 0.2050 & 0.2472 & 0.2115 & 0.1892 & 0.1472\\ 0.1690 & 0.2173 & 0.2570 & 0.1987 & 0.1580\\ 0.1793 & 0.2050 & 0.2240 & 0.2107 & 0.1811 \end{array}] \end{array}$$ (25)

$$\begin{array}{c} M_{U}=[\begin{array}{ccccc} 0.2149 & 0.2165 & 0.2106 & 0.2019 & 0.1561\\ 0.1513 & 0.1946 & 0.2129 & 0.2429 & 0.1983\\ 0.1502 & 0.1931 & 0.2394 & 0.2348 & 0.1826\\ 0.1629 & 0.2094 & 0.2366 & 0.2085 & 0.1825 \end{array}] \end{array}$$ (26)

A comprehensive evaluation matrix is shown below:

$$\begin{array}{c} B_{P}=[\begin{array}{ccccc} 0.1934 & 0.2163 & 0.2205 & 0.1993 & 0.1706 \end{array}] \end{array}$$ (27)

$$\begin{array}{c} B_{S}=[\begin{array}{ccccc} 0.1872 & 0.2260 & 0.2326 & 0.1955 & 0.1588 \end{array}] \end{array}$$ (28)

$$\begin{array}{c} B_{U}=[\begin{array}{ccccc} 0.1723 & 0.2039 & 0.2244 & 0.2211 & 0.1783 \end{array}] \end{array}$$ (29)

Therefore, according to Eq 23, the three-dimensional integrated assessment values are as follows:

$$\begin{array}{c} Z_{P}=5.1257,Z_{S}=5.1751,Z_{U}=4.9416 \end{array}$$ (30)

Finally, combining the combined risk values of the three dimensions, we can arrive at a final risk value of *Z*_*P*_ = 5.1257, *Z*_*S*_ = 5.1751 and *Z*_*U*_ = 4.9416 implying a value of 5.0808 for the hydrogenation station PPP project.

Although the level of uncontrollability is moderate, the risks of both likelihood and consequence fall between moderate and high. Therefore, overall risk level of hydrogen refueling station infrastructure PPP projects is moderate. Regarding risk management criteria, appropriate steps can be taken to mitigate and control risk factors. Throughout the lifespan of the project, it is imperative to not only effectively plan for risk management, but also to minimize any potential loss.

## Sensitivity analysis

Sensitivity analysis is designed to avoid risk scores being affected by differences in the number of experts and how much emphasis they place on the opinions of the risk takers. In practice, the attitude of the expert may also change with the environment and other factors.

We perform stability tests by changing the risk weights. The risk weights were divided into the following three groups: (1) Model A adopted the original risk weights; (2) The weight of module B is increased by 10%; (3) 10% weight reduction for module C. The risk values changed as the risk factor weights were increased (as shown in Table 8). This demonstrates how the conclusions of the risk assessment were impacted by the experts’ varied viewpoints. No matter how the weights were altered, the total risk value fluctuated between 1.00% and 2.00%. It is worth noting that in Model B, the change in weighting for C13 and C2 resulted in a higher change in risk value than the other risk factors. Therefore, participants should place a high priority on these risks. In addition, in Model C, the value at risk decreases sharply as the weights of C1 and C2 are reduced.

**Table 8:** Results with different model.

	B				C			
	P	S	U	Z	P	S	U	Z
C1	5.1877	5.2355	4.9381	5.1187	5.0560	5.1065	4.8120	4.9898
C2	5.1951	5.2445	4.9396	5.1246	5.0547	5.1043	4.8138	4.9893
C3	5.1728	5.2254	4.9272	5.1068	5.0770	5.1234	4.8262	5.0071
C4	5.1528	5.2016	4.9056	5.0850	5.097	5.1472	4.8478	5.029
C5	5.1449	5.1943	4.8949	5.0763	5.1049	5.1545	4.8585	5.0376
C6	5.1440	5.1936	4.8941	5.0755	5.1058	5.1552	4.8593	5.0384
C7	5.1580	5.2105	4.9093	5.0909	5.0918	5.1383	4.8441	5.0231
C8	5.1921	5.2371	4.9360	5.1200	5.0578	5.1117	4.8174	4.9940
C9	5.1893	5.2365	4.9345	5.1184	5.0605	5.1123	4.8189	4.9956
C10	5.1625	5.2094	4.9100	5.0922	5.0874	5.1394	4.8434	5.0217
C11	5.1783	5.2245	4.9240	5.1072	5.0715	5.1243	4.8294	5.0068
C12	5.1518	5.2011	4.9018	5.0832	5.0981	5.1477	4.8516	5.0308
C13	5.1938	5.2423	4.9414	5.1241	5.0622	5.1133	4.8153	4.9952
C14	5.1869	5.2354	4.9357	5.1176	5.063	5.1134	4.8177	4.9963
C15	5.1621	5.2099	4.9112	5.0927	5.0877	5.1389	4.8422	5.0212
C16	5.1591	5.2070	4.9099	5.0903	5.0908	5.1418	4.8435	5.0236

**Fig 7:**
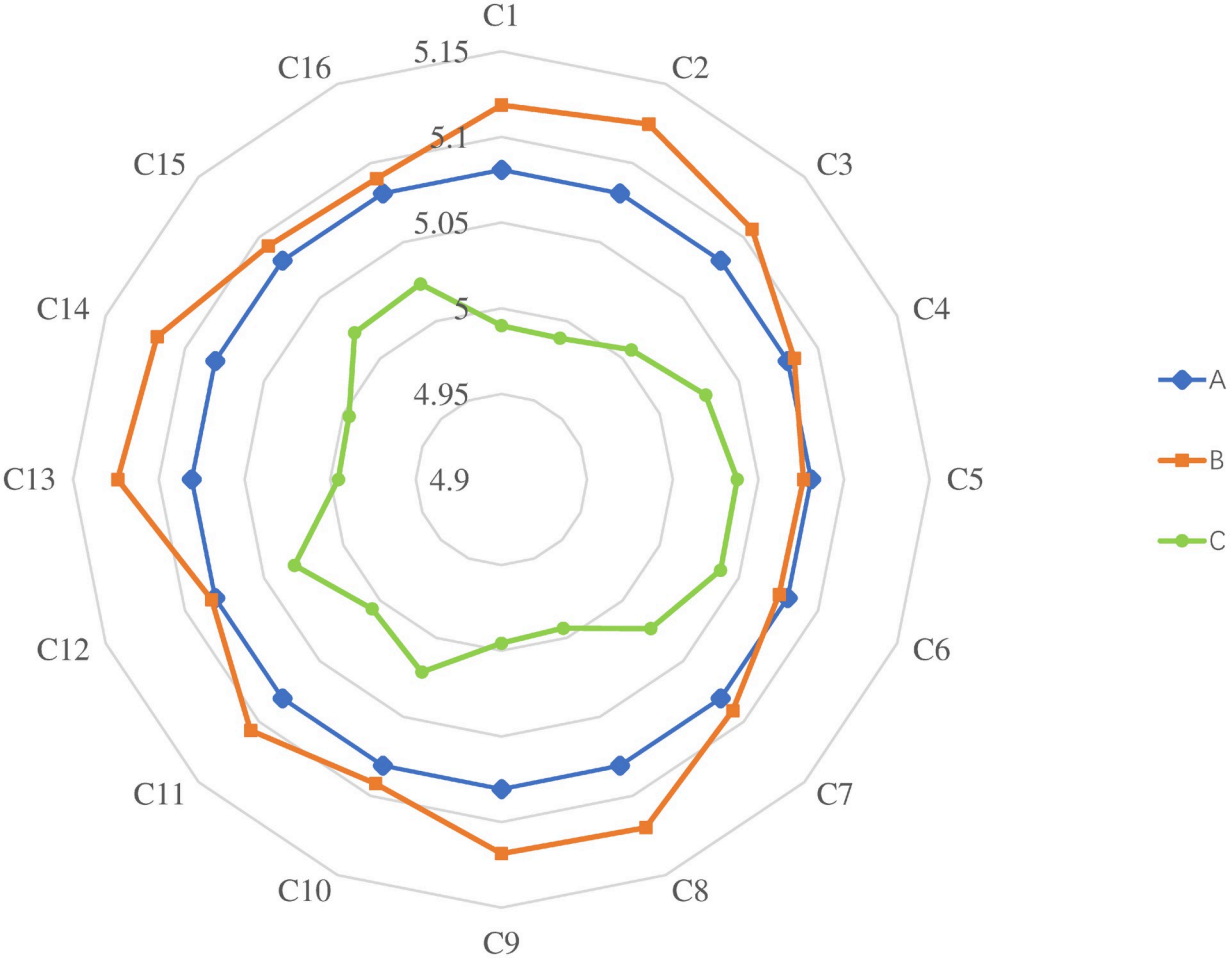
Comparison of three models.

As shown in Fig 7, the risk values changed accordingly as the risk factor weights were adjusted, demonstrating the impact of different viewpoints of the experts on the conclusions of risk assessment. The changes in weights for C13 and C2 caused a greater impact on the risk value in Model B than other risk factors, indicating that more attention should be paid to these risks by participants. In addition, in Model C, the risk value decreased significantly when the weights of C1 and C2 were reduced. On the other hand, Model C’s scope and probability grew dramatically with the increase of the weight of C2, implying that potential considerable losses need to be prevented proactively.

## Risk response

As shown in the risk assessment section, the construction of Chinese Hydrogenation Station construction project is faced with certain challenges due to the relatively high risk. A key component in integrated risk management is to develop countermeasures to reduce such risks. After potential risks and threats to which the hydrogenation station may be exposed have been identified and quantified, and the associated life cycle vulnerabilities have been evaluated, necessary measures should be taken to protect the entire investment against internal and external threats. The principles of risk avoidance, risk reduction, risk absorption, and risk transfer should serve as the foundation. Risk-sharing suggestions are listed in Table 9, which provides responses and recommendations for risk-sharing parties, and can be used as a reference for both the government and the private sector.

**Table 9:** PPP hydrogenation station risk sharing.

Risk factor	Government	Private	Sharing
Stability and continuity of local policies	✓		
Industry standards and market access risks	✓		
Government decision risk	✓		
Land acquisition	✓		
Inflation risk			✓
Interest rate change			✓
Capital supervision is not perfect			✓
Hydrogenation station design risk		✓	
Unreasonable layout		✓	
Possible future payment risks		✓	
High operating and maintenance costs		✓	
Risk of hydrogenation station completion			✓
Lack of PPP experience			✓
Unclear delineation of obligations and responsibilities			✓
Government credit	✓		
Third party fault			✓

### Government

- **Stability and continuity of local policies**. The government should clarify relevant policies, such as financial subsidy mechanisms. One of the reasons for the current incomplete construction of hydrogenation stations in China is that the lack of policies on hydrogenation stations makes them expensive to build and operate.
- **Land acquisition**. There should be clearer policies on site planning, the nature of land, construction approval and construction subsidies for hydrogenation stations. During the planning process, consideration should be given to the construction of hydrogenation stations to obtain suitable locations.
- **Industry standards and market access**. They are important considerations in hydrogenation station projects, as they ensure the safe and reliable operation of the stations and facilitate the deployment and adoption of hydrogen fuel technologies.
- **Government decision risk**. Before a project can begin, with a win-win goal in mind, the government may bargain with the private sector to decide the project’s condition. In addition, preconditions such as approval requirements must be established to avoid lengthy decision-making.
- **Government credit**. The government departments shall make appropriate representations and guarantees in the transfer agreement. In addition, the government may enter into agreements to share profits with the private sector mechanisms.

### Private sectors

- **High operating and maintenance costs**. Since hydrogen is flammable and explosive, the hardware facilities required for the construction of a hydrogenation station are very demanding. As a result, hardware costs increase and operating costs become more difficult to amortize. Accurate cost estimation before a project is put into operation can address these challenges.
- **Unreasonable layout**. Poor layout can result in low efficiency, leading to waste of resources and financial loss. Therefore, the private sector should take appropriate measures before making informed decisions.
- **Hydrogenation station design risk**. The private sector should strengthen its technical research. Mainly for the main technical equipment of hydrogenation station.
- **Possible future payment risks**. On one hand, a business should establish a partnership with a third-party company. On the other hand, it is possible to create a secure and practical payment network. The business can work with other companies to expedite the search process.

### Risk sharing

- **Inflation risk**. Inflation directly results in higher project costs. To address this risk, an adjustment factor should be established before the project starts to deal with this volatility. One such method is for participants to reference local prices.
- **Interest rate change**. The impact of interest rates can be reflected in changes to finance charges. For the government, one solution may be to change the interest rate in the formula for pricing adjustments. The private sector, however, financial instruments can be used reasonably.
- **Capital supervision is not perfect**. The two sides should work together to improve the capital chain’s management, such as disbursement, reimbursement and funding approval processes. In addition, it is effective to establish a monitoring system to enable dynamic management.
- **Lack of PPP experience**. Once the government selects the private sector partner, it should carefully consider whether chosen party has prior experience in PPP projects. For the private sector, it is crucial to learn from the lessons of previous similar projects before embarking on any new PPP endeavor. By doing so, both parties can ensure a smooth and successful collaboration.
- **Unclear delineation of obligations and responsibilities**. The concession agreement should clearly specify the responsibilities and obligations of the parties involved. In the case of any breach of contract by one party, the aggrieved party shall have the right to claim damages or compensation, based on the terms and conditions set forth in the agreement.
- **Third party fault**. In concession agreements, it is necessary for both governments and the private sector to include provisions that grant them the authority to rectify projects or file claims against third parties.
- **Hydrogenation station completion risk**. After the installation of hydrogenation stations, participants should appropriately plan the development of infrastructure for hydrogen refuelling while taking into account risk factors in advance. It is crucial to strengthen research related to the hydrogen supply chain.

## Conclusion

To effectively tackle the formidable task of carbon reduction and balance the potential benefits and risks in the development of hydrogen energy in the transportation sector, a risk management framework has been proposed for the management of public-private partnership (PPP) hydrogen refueling station projects. The framework identifies operating revenues, costs, industry standards, and technical risks as areas requiring special attention. Countermeasures have also been proposed based on the evaluation results. The government should play a pivotal role in setting policies and standards, while the private sector should focus on enhancing technological innovation to further boost the development of hydrogen energy.

This study can serve as a reference for participants to better handle risks, thus facilitating the successful completion of hydrogen refueling station PPP projects. The 3D framework employed in this paper is highly comprehensive in terms of assessing the probability, scope, and uncontrollability of risks in such construction projects. Moreover, a combined weighting approach is adopted to avoid one-sidedness in subjective and objective assignments, thus enabling a more accurate evaluation of the index weights. Based on this, the ultimate goal of this paper is to propose constructive suggestions for revalidating risk indicators through sensitivity analysis.

However, further research is required to investigate effective concessions and pricing mechanisms, in order to continuously enhance the profitability model.

## Supporting information

S1 File Raw expert scoring data. (XLSX)

S2 File Range analysis dimensionless matrix. (XLSX)
